# Chemical, Clinical and Histomorphometric Comparison between Equine Bone Manufactured through Enzymatic Antigen-Elimination and Bovine Bone Made Non-Antigenic Using a High-Temperature Process in Post-Extractive Socket Grafting. A Comparative Retrospective Clinical Study

**DOI:** 10.3390/dj7030070

**Published:** 2019-07-01

**Authors:** Danilo Alessio Di Stefano, Terry Zaniol, Lorenzo Cinci, Laura Pieri

**Affiliations:** 1 Dental School, Vita-Salute University IRCCS San Raffaele, Via Olgettina 58, 20132 Milan, Italy; 2 Private Practice, Crocetta del Montello, 31035 Treviso, Italy; 3 Department of Neuroscience, Psychology, Drug Research and Child Health (Neurofarba), Pharmacology and Toxicology Section, University of Florence, Viale G. Pieraccini 6, 50139 Florence, Italy; 4 Department of Health Sciences, Interdepartmental Forensic Medicine Section, University of Florence, Viale G. Pieraccini 6, 50139 Florence, Italy

**Keywords:** post-extractive sockets, xenograft, bone formation

## Abstract

Enzyme-deantigenic equine bone (EDEB) and anorganic bovine bone (ABB) are two xenografts made non-antigenic through different processing methods. This study aimed to characterize them for the presence of native bone collagen and other proteins and to compare their histomorphometric outcome when they were used to graft post-extractive sockets. The records of 46 patients treated with EDEB (*n* = 22) or ABB (*n* = 24) and followed-up for at least four months after delayed implant placement, were retrospectively collected. Samples of EDEB and ABB were analyzed using Attenuated Total Reflection Fourier Transform Infrared and Sodium Dodecyl Sulfate-Polyacrylamide Gel Electrophoresis for the presence of collagen and other proteins. For histomorphometric analysis on bone specimens, newly formed bone and residual biomaterial percentages were calculated. Results of the present study show that EDEB contains type I bone collagen in its native conformation, while no proteins were detected in ABB. Grafting EDEB resulted in a significantly greater quantity of newly formed bone and less residual biomaterial. Our findings suggest that the manufacturing process can greatly affect the graft behavior and a process preserving collagen in its native form may favor bone tissue regeneration.

## 1. Introduction

Following tooth extraction, the alveolar bone at the post-extractive sockets undergoes resorption according to a well-studied temporal and spatial pattern [1,2]. If implant placement cannot be performed immediately at the time of extraction, the alveolar bone may resorb to the point that implant-supported prosthetic reconstructions cannot achieve optimal functional and aesthetic results anymore [1,3]. To limit bone resorption, the surgeon may graft the socket with a bone substitute according to the principles of socket preservation [4]. Autogenous bone is regarded as the gold standard grafting material, because it contains bone cells (osteoblasts, osteocytes, stem cells) and growth factors that favor bone regeneration [5]. However, using autogenous bone requires additional surgery and increases both morbidity and the risk of intra- and post-surgical complications [6]. Alternative bone grafts may be used, either allogeneic, synthetic, or natural [7,8]. According to their degradation, bone repair materials can be classified into bio-inert and biodegradable [8]. Bio-inert materials are mostly inert implants that stay in the human body forever until removed. Biodegradable materials are preferred as once bone repair and healing has occurred, their removal via in vivo degradation leads to better clinical performance and a biomechanical bone behavior [8]. Among biodegradable materials, natural grafts may be a viable option [8] and, among these, bone xenografts are regarded as particularly promising because of the similarities between human and animal (mammal, non-human) bone architecture and composition [9]. Anorganic bovine bone (ABB) is the xenograft having the longest history of clinical use for bone augmentation in oral and maxillofacial surgery [10,11]. ABB is obtained by processing bovine bone using high temperatures to eliminate all antigens and proteins [12]. When human osteoclasts were cultured on ABB slices, they adhered less and showed less absorptive capacity than when they were cultured on raw bovine bone slices used as a control [13]. Several clinical studies involving histomorphometric analyses have shown that ABB has a long resorption time, with residual particles persisting in the grafted sites even years after grafting [14,15,16]. ABB is made of cancellous bovine bone, and its granule size is 0.25–1 mm [10,11]. No data concerning its porosity is publicly available. An alternative xenograft is enzyme-deantigenic equine bone (EDEB) made non-antigenic using digestive enzymes. EDEB is a 1:1 mixture of cancellous and cortical bone, and its granule size is 0.5–1 mm. Even for EDEB, no data concerning its porosity is publicly available. EDEB has already been used in peri-apical cyst-removal management, periodontal defect correction, and horizontal and vertical ridge and sinus augmentation [17,18,19,20,21,22,23,24]. It was also shown to regenerate bone in orthopedic applications [25]. In previous investigations on EDEB, it was stated that the enzymatic process used for its manufacturing preserves type I bone collagen in its native state [13,17,19,25]. Bone collagen is involved in or modulates many processes related to bone regeneration [26,27,28,29]. When human osteoclasts were cultured on EDEB slices as was previously done on ABB [13], they showed a greater adhesion and resorption activity than they did on ABB [30], possibly because of the presence of native collagen in EDEB [30].

In a randomized clinical trial on 40 patients that compared the use of EDEB and ABB for maxillary sinus augmentation, grafting EDEB resulted in a greater quantity of newly-formed bone (NFB), and a smaller quantity of residual biomaterial (RB) at implant insertion than when ABB was used [31]. A retrospective study investigated bone formation over time following maxillary sinus augmentation using EDEB and showed that new bone formation occurred at an early time (<3 months) after grafting [32]. Both the in vitro and in vivo remodeling behavior of EDEB were explained by the claim that it contains type I bone collagen in its native state. This claim, yet, has not been proved—to the author’s knowledge—in any previous publication concerning EDEB. Additionally, no literature data exist comparing the histomorphometric outcome of ABB and EDEB when they were used to graft post-extractive sockets. The aim of the present investigation was to assess whether EDEB contains collagen in its native state by Attenuated Total Reflection Fourier Transform Infrared (ATR-FTIR) and Sodium Dodecyl Sulfate-Polyacrylamide Gel Electrophoresis (SDS-PAGE) analysis, and to retrospectively compare histomorphometric data relating to bone samples collected from post-extractive sockets that had been grafted using EDEB and ABB.

## 2. Materials and Methods

### 2.1. ATR-FTIR Spectroscopy

A minimum quantity of either EDEB (Osteoplant Osteoxenon, Bioteck, Vicenza, Italy) or ABB (Bio-Oss, Geistlich Pharma, Wolhusen, Switzerland) was loaded into the ATR module of an ATR-FTIR spectrometer (Cary 630, Agilent, Santa Clara, CA, USA) and a FTIR spectrum was acquired within a 4000–650 cm^−1^ range with a 2 cm^−1^ resolution. The sample and the background were scanned, respectively, 30 times and 16 times. A qualitative analysis of the spectra was then performed by comparing the wavenumber of the most significant peaks with that of a reference wavenumber library, in order to identify the main functional molecular groups.

### 2.2. SDS-PAGE Analysis

Between 1 and 2 g of the sample of interest were completely demineralized by overnight immersion in 0.5 M HCl (Sigma-Aldrich, Milano, Italy), dialyzed using Milli-Q water (Simplicity, Merck Millipore, Vimodrone, Italy) and lyophilized. Then, 50 mg were later solubilized in 1 mL 0.1 M acetic acid (Sigma-Aldrich) for 4 h at room temperature, using a thermoshaker set at 400 rpm (PHMT, Grant Instruments, Shepreth, UK). The extracted samples and a collagen standard (PureCol-S Bovine Collagen, Advanced Biomatrix, San Diego, CA, USA) were then diluted 1:10 using an electrophoresis buffer (NuPage LDS Sample Buffer 1X, Invitrogen, Rodano, Italy) containing a reducing agent (NuPage Reducing Agent 1X, Invitrogen). The solution was then kept for 10 min under shaking at 400 rpm and 70 °C using a thermoshaker (PHMT, Grant Instruments). Electrophoresis was carried out by loading an electrophoresis gel (NuPage Bis-Tris Mini Gels 10 Well, Invitrogen, ThermoFisher Scientific, Waltham, MA, USA) with the samples of interest together with a molecular weight marker (Precision Plus Protein Standard Dual Color, Bio-Rad, Segrate, Italy). The gel was run in a running buffer (MOPS SDS 1X, Invitrogen) using a 200 V electric field for 50 min. Staining followed according to a standard protocol using colloidal Coomassie Stains (Bio-Rad).

### 2.3. Retrospective Data Assessment

Clinical records were selected among those of patients who presented to three private practices in Italy, between January 2008 and December 2011, seeking implant-supported rehabilitation. Patients included in the present retrospective study (1) underwent one or more atraumatic tooth extraction anywhere in the maxillae; (2) had no acutely infected sockets; (3) had sockets walls intact; (4) had their post-extractive sockets immediately grafted using either EDEB or ABB; (5) had delayed implant placement carried out between 4 and 8 months after grafting; and (6) had at least one biopsy specimen collected at the grafted site at the time of implant placement. Other inclusion criteria were: an age between 18 and 70 years and the lack of any systemic diseases. Patients were eligible for regenerative treatment if they did not present any of the following: pregnancy; osteoporosis, neoplasia, or psychiatric disease; acute oral infections; coagulation disorders; history of chemotherapy or radiotherapy in the head or neck region; immunocompromised status; current bisphosphonate therapy; chronic alcohol or drug abuse; or smoking more than 10 cigarettes per day. All patients provided their informed consent to the treatment and to the use of their clinical data for retrospective collection and analysis. Being retrospective in nature, no Ethic Committee approval was searched for the present study. All patients underwent clinical examination and radiographic assessment through orthopantomography (OPT) or intraoral radiographs. Cone-beam Computed Tomography scans were acquired only if needed.

### 2.4. Surgical Procedure

Antibiotic prophylaxis (amoxicillin/clavulanic acid, Augmentin, Glaxo-SmithKline, Verona, Italy) (2 g, 1 h before surgery and then every 12 h for 8–10 days) was initiated, and patients were subjected to mouth rinses with chlorhexidine 0.2% (Corsodyl, Glaxo-SmithKline), to be continued for two weeks after surgery. Nimesulide 100 mg (Aulin, Roche, Milano, Italy) also was administered 1 h before surgery and then twice a day for seven days. The surgical area was anesthetized with articaine hydrochloride 40 mg/mL and adrenaline 1:100,000. No flaps were elevated, and the compromised tooth was extracted atraumatically. After careful socket debridement using manual instruments (Lucas Curettes, Hu-Friedy, Chicago, IL, USA), the socket was grafted either using EDEB or ABB. To do that, EDEB or ABB particles were hydrated with sterile saline and inserted into the cavity, applying gentle pressure to stabilize them. The amount of material used in all patients was sufficient to achieve complete socket filling. After gently detaching the gingival rim from the underlying bone, a collagen membrane (Biocollagen, Bioteck, Vicenza, Italy) was inserted below the gingival margins. These were stabilized with a single cross stitch, using non-resorbable 5.0 sutures. Sutures were removed after 10 days. Patients were subsequently controlled once a month. When intra-oral radiographs showed that the graft had significantly changed its radio-opacity, suggesting significant remodeling, implant surgery was performed as follows: after antibiotic prophylaxis and anesthesia as in the extractive surgery, a full-thickness flap was raised, the bone was inspected, and biopsy samples were obtained from the occlusal aspect of the alveolar ridge using a trephine drill under irrigation with sterile saline. All biopsies were 3 mm in diameter and 10 mm in length and were marked on the occlusal side for orientation during histologic processing. Titanium implants, 3.75 to 5.0 mm wide and 10 to 14 mm long, were placed at the biopsy sites. Three months after placement, the implants were uncovered, and healing screws were attached. Three weeks later, a radiograph was taken, and a dental impression was made with pick-up impression copings in order to manufacture a provisional prosthesis that was delivered after 10 days. Patients wore this for approximately 40 days, at which point the definitive abutments and metal-ceramic crowns were delivered. Patients followed a maintenance program comprising professional oral hygiene every 6 months, for a follow up period variable from patient to patient.

### 2.5. Histology and Histomorphometry

Each biopsy was placed in a test tube containing buffered 10% formalin. The tube was then marked with a unique alphanumeric code that could not be related to the patient or the xenograft and sent to the histologists (LC, LP) who were, therefore, unaware of the material used for grafting each sample. Bone cores were decalcified for 21 days in a 0.76 M sodium formate and 1.6 M formic acid solution (Panreac Quimica, Barcelona, Spain). The sample was subsequently dehydrated in ascending concentrations of ethanol and embedded in paraffin. This procedure aimed at achieving rapid tissue infiltration with only minimal sample shrinkage, in order to provide a sample morphology still highly representative of the in vivo bone features.

The bone cores were cut into 5 μm-thick sections, mounted on slides, and stained with hematoxylin-eosin. One assessor (LC) provided, for each sample, a qualitative report aimed at identifying any sign of inflammatory or immune reactions and provided a general qualitative assessment. Slides were also observed under polarized light to assess the presence of lamellar (mature), mineralized bone. Morphometrical measurements were performed on digital photomicrographs collected at 10 × magnification. Each whole sample image was analyzed independently by two of the authors (LC, LP) using the Image J 1.33 analysis software (National Institute of Health, Bethesda, USA). For each image, the total sample area (TSA), the total bone area (TBA), the newly formed bone area (LBA) and the residual bone substitute area (RBA) were measured. Each of the two assessors repeated the assessment in triplicate. Average newly formed bone (NFB) and residual biomaterial (RB) were then calculated and expressed as the percentage over the total sample area (%NFB = LBA × 100/TSA; %RB = RBA × 100/TSA).

### 2.6. Statistical Analyses

NFB and RB are given as the mean percentage of all sections. Differences between groups in relation to age, healing period duration, NFB and RB were investigated through a two-tailed *t* test. To compare NFB and RB, the grafted socket was regarded as the statistical unit of analysis. Differences were regarded as significant if *p* < 0.05. A dedicated software program (Origin 9.0, Microcal, Northampton, MA, USA) was used for all statistical analyses. All values are presented as mean ± standard deviation (SD).

## 3. Results

### 3.1. ATR-FTIR Spectroscopy Assessment

**Figure 1:**
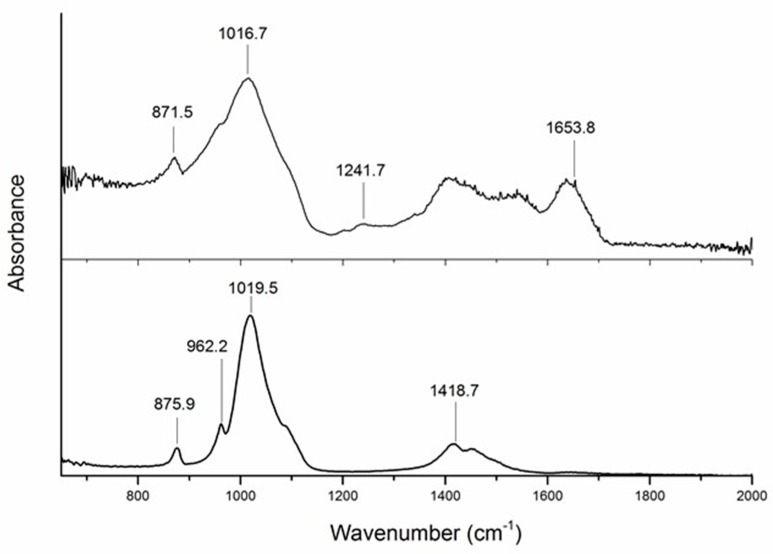
Attenuated Total Reflection Fourier Transform Infrared (ATR-FTIR) spectra of EDEB (top) and anorganic bovine bone (ABB) (bottom). Both plots show peaks corresponding to carbonates and phosphates (871.5 and 1016.7 cm^−1^ for EDEB; 875.9, 1418.7, 962.2 and 1019.5 cm^−1^ for ABB). Yet, peaks corresponding to amidic bonds of proteins (Amide III, 1241.7 cm^−1^ and Amide I, 1653.8 cm^−1^) can be observed only in the EDEB plot.

ATR-FTIR spectra corresponding to EDEB and ABB are shown in Figure 1. The plot corresponding to the ABB sample showed characteristic peaks at 875.9 and 1418.7 cm^−1^, corresponding to different vibration modes of carbonate, and at 962.2 and 1019.5 cm^−1^, corresponding to different vibration modes of phosphate. Significantly, the plot showed no peaks corresponding to amidic groups, indicating that—within the limits of detection of this technique—no collagen or other proteins are present in ABB. The plot corresponding to the EDEB sample showed again peaks corresponding to carbonates (871.5 cm^−1^) and phosphates (1016.7 cm^−1^), but also peaks corresponding to the amidic bonds of proteins (Amide III, 1241.7 cm^−1^ and Amide I, 1653.8 cm^−1^).

### 3.2. SDS-PAGE Analysis

**Figure 2:**
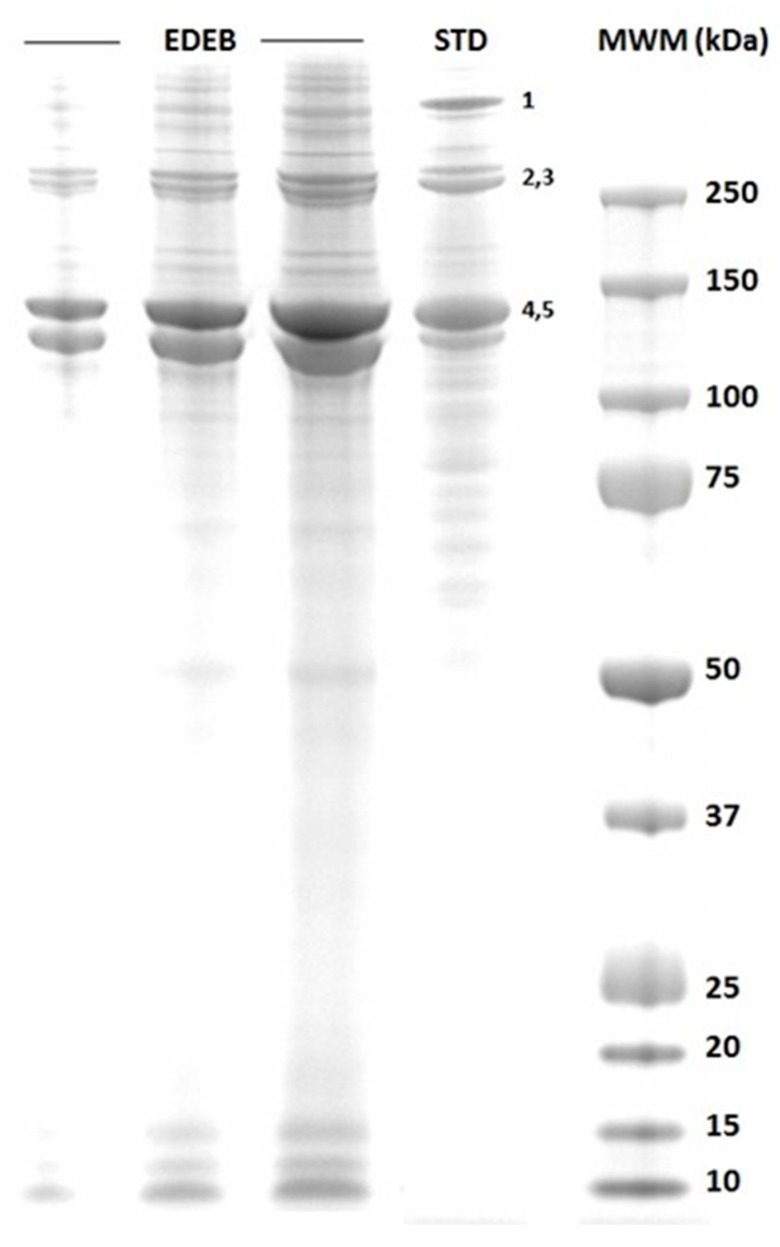
Sodium Dodecyl Sulfate-Polyacrylamide Gel Electrophoresis (SDS-PAGE) on an EDEB sample. The first three lanes (EDEB) correspond to different quantities of sample loaded into the gel (2, 5, 10 µL respectively). The fourth lane (STD) corresponds to the bovine collagen standard. Molecular Weight Markers (MWM) are shown in the last lane. Band 1 corresponds to the collagen 2α_1_1α_2_ trimer having a MW equal to 397 kDa; bands 2 and 3 correspond respectively to the α_1_α_2_ and to the α_1_α_1_ dimers, whose MW is respectively 268 and 258 kDa. Bands 3 and 4 correspond to the single α_2_ and α_1_ collagen subunits having a MW equal to 139 and 129 kDa respectively. All EDEB lanes show all 1–5 bands, corresponding to native collagen, and few other bands possibly corresponding to collagen degradation products. The intensity of 1–5 bands in EDEB lanes, much higher than that of all the other bands, indicates that most, if not all, collagen is in its native conformation.

As ATR-FTIR analysis showed no proteins were present in ABB, SDS-PAGE was performed on an EDEB sample only. The corresponding electrophoretic pattern is shown in Figure 2. All the bands corresponding to the trimeric 2α_1_1α_2_ and the dimeric α_1_α_2_, α_1_α_1_ forms of collagen as well as those of the separate α_2_ and α_1_ collagen subunits were observed in the migration pattern, and all presented a high intensity. Few, low-intensity bands were also present, possibly corresponding to collagen degradation products. These observations indicate that EDEB contains most, if not all, collagen in its native, not-degraded conformation.

### 3.3. Histology and Histomorphometry

Histomorphometric results of the present study are summarized in Table 1. Records retrieved related to 46 patients, including 25 men and 21 women with a mean age of 54 (range 43 to 75). Patients whose sockets were grafted using EDEB were 22, 11 men and 11 women, having a mean age of 52.8 ± 5.9 years (range 43 to 65). Patients grafted using ABB were 24, 14 men and 10 women; their mean age was 55.0 ± 8.1 years (range 44 to 73). The two groups were not significantly different as far as the age of the patients was concerned (*p* = 0.29).

**Figure 3:**
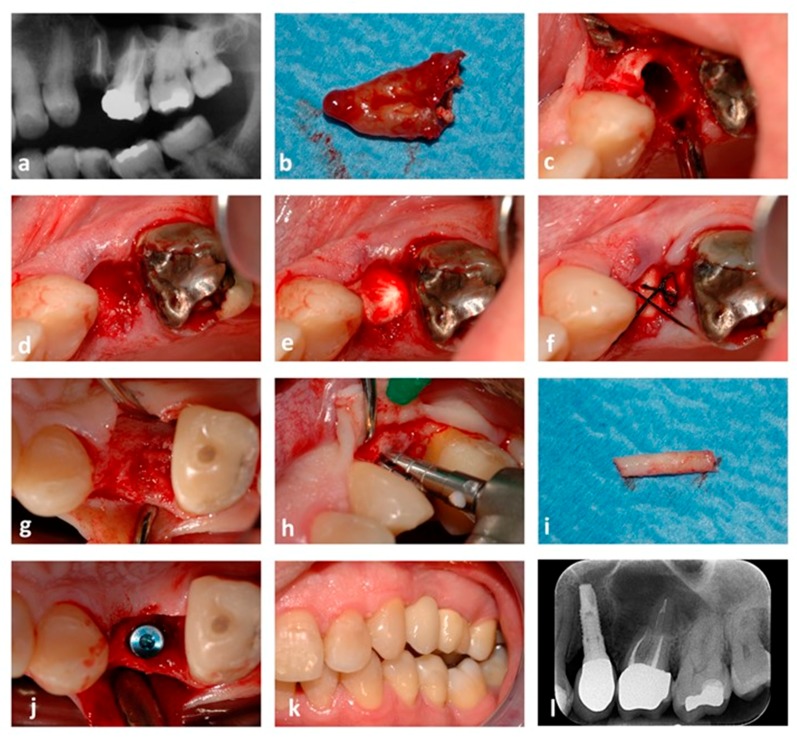
Post-extractive socket grafting. A case where EDEB was used is shown. (**a**) X-ray showing the initial patient status. Tooth 25 is fractured and compromised; (**b**) the element after being extracted; (**c**) the post-extractive socket before grafting; (**d**) the socket after being grafted; (**e**) a collagen membrane is placed below the gingival rims to cover the graft; (**f**) a single cross stitch stabilizes the reconstruction; (**g**) appearance of the regenerated socket 4.1 months after the grafting surgery; (**h**) a bone core is collected using a trephine bur; (**i**) the bone sample after collection; (**j**) an implant (Stone, 3.75 × 14 mm, IDI Evolution, Concorezzo, Italy) is placed into the regenerated bone; (**k**) the final prosthetic rehabilitation; (**l**) one-year control radiograph, showing maintenance of peri-implant bone levels.

**Figure 4:**
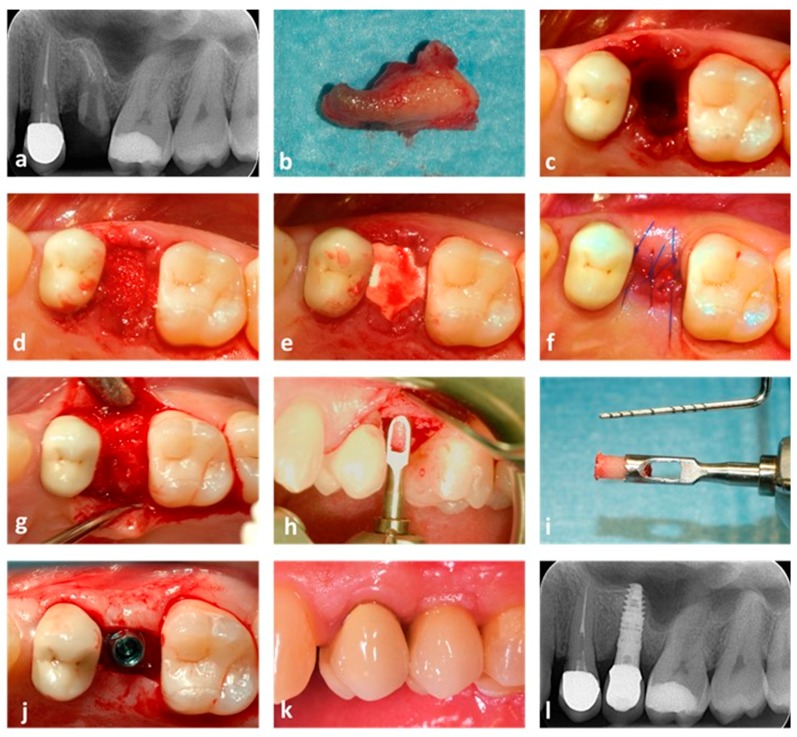
Post-extractive socket grafting. A case where ABB was used is shown. (**a**) X-ray showing the initial patient status. Tooth 25 is fractured and compromised; (**b**) the element after being extracted; (**c**) the post-extractive socket before grafting; (**d**) the socket after being grafted; (**e**) a collagen membrane is placed below the gingival rims to cover the graft; (**f**) single cross stitches stabilize the reconstruction; (**g**) appearance of the regenerated socket 5.3 months after the grafting surgery; (**h**) a bone core is collected using a trephine bur; (**i**) the bone sample after collection; (**j**) an implant (Stone, 3.75 × 14 mm, IDI Evolution, Concorezzo, Italy) is placed into the regenerated bone; (**k**) the final prosthetic rehabilitation; (**l**) one-year control radiograph, showing maintenance of peri-implant bone levels.

All patients completed the healing period following the post-extractive socket grafting procedure with no complications. The mean healing period of patients grafted using EDEB and ABB was 4.1 ± 1.2 and 4.4 ± 1.2 months respectively, the two being not significantly different (*p* = 0.37). Radiographs (OPTs or intraoral radiographs) taken over the follow up control before implant placement showed proper integration of the bone grafts in all patients. Figure 3 and Figure 4 show two representative cases.

### 3.4. Qualitative Histologic Analyses

**Figure 5:**
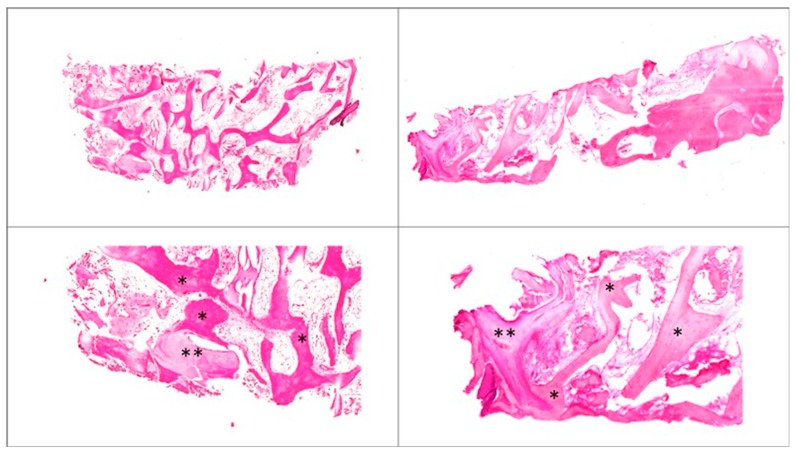
Hematoxylin-eosin staining, histology. **Top**: 3.5× magnification. **Left**, an ABB case. **Right**, an EDEB case. **Bottom**: detail, 10× magnification. Symbols: *, newly formed bone; **, residual biomaterial.

Samples from both groups were characterized by extensive newly-formed bone areas (Figure 5). Bone graft particles could be observed in both groups, and were characterized by their affinity for hematoxylin and for having bone lacunae devoid of osteocytes. Due to their animal origin, their aspect resembled human bone, with osteocytes eliminated by the antigen elimination process. A close contact between newly formed bone and residual particles was observed in samples from both groups. No inflammation signs nor cartilage-like tissue were observed in any sample. Active osteoblasts were observed lining both the bone graft and the newly regenerated bone in all samples. Mineralized bone was present in most samples from both groups.

### 3.5. Histomorphometric Results

**Figure 6:**
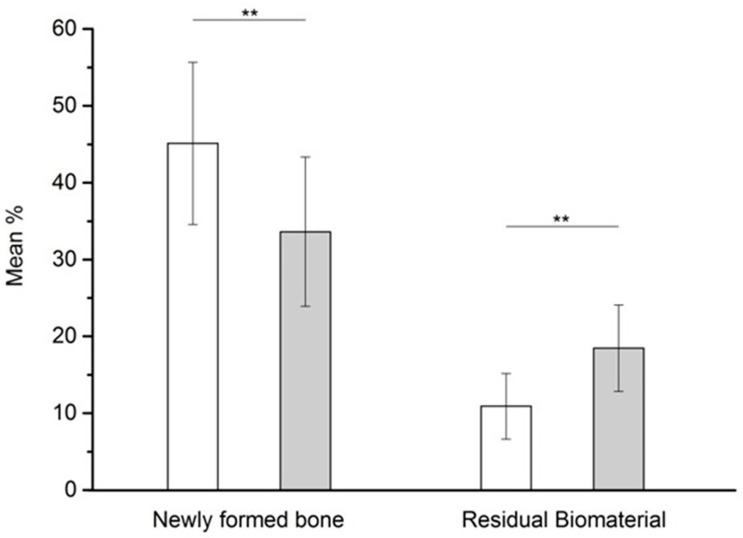
Histomorphometric analysis results. Difference between EDEB (white) and ABB (light gray) are significant for both newly formed bone (NFB) and residual biomaterial (RB) at a *p* < 0.05 level of confidence (**).

Histomorphometric results were NFB = 45.12% ± 10.54%; RB = 10.91% ± 4.27% (*n* = 41 sockets) for EDEB, and NFB = 33.61% ± 9.71%; RB = 18.47% ± 5.62% (*n* = 43 sockets) for ABB (Table 1). The difference between the two groups was significant, at a 0.05 level of confidence both for NFB and RB (Figure 6).

## 4. Discussion

Results of the present study show that EDEB contains type I bone collagen in its native conformation. To the author’s knowledge, this is the first demonstration of this property of EDEB. Such feature of EDEB, in fact, was claimed in previous clinical papers concerning its use in bone regeneration surgeries, but no proof had been provided. This result confirms that the enzymatic processing method applied to make equine bone non-antigenic is selective, allowing to preserve bone collagen unaltered. Results concerning ABB are consistent with previous literature on its residual protein content and with the expected outcome of a high temperature (>600 °C) processing method, aimed to totally destroy any organic molecules [12].

At histologic examination, residual ABB and EDEB particles were observed to be fully integrated with the surrounding, newly regenerated bone tissue. No inflammation was present, indicating full biocompatibility of both grafts. These observations are consistent with previous in vitro and in vivo studies on EDEB [17,33].

Concerning the histomorphometric results of the present investigation, a significantly higher amount of newly formed bone at the time of implant placement was observed in sites grafted with EDEB than in those grafted with ABB. Furthermore, a greater amount of residual biomaterial was observed in the ABB sites. Previous studies concerning the resorption of ABB when used for socket filling show NFB varying from a minimum of 19.4% to a maximum of 63.9–69.1% at 4, respectively, and 9–12 months after grafting [34,35]. When measured at 6 months after grafting, NFB varied from 31.4% to 39.4% [35,36,37], that is between values that are consistent with the results of the present studies.

The greater amount of NFB observed when EDEB was grafted may partially be explained by the different behavior of osteoclasts that was observed when they were cultured on the two materials in previous in vitro experiments [13,30]. This behavior may, in turn, be explained by the presence of native collagen in EDEB and its absence in ABB. More extensive studies should be performed to investigate if the two processing methods also differently affect the chemical-physical properties of the bone mineral component. Indeed, a processing method involving high temperatures as that used to manufacture ABB should also be expected to modify the composition and the crystalline structure of bone apatite; this might be assessed, for example, using X-ray diffraction (XRD) techniques. In the authors’ opinion, the different histomorphometric behavior is unlikely to be caused by the difference in particle size (0.25–1 mm of ABB vs. 0.5–1 mm of EDEB), or by the different type of the bone of origin (cancellous only for ABB, both cancellous and cortical for EDEB). Indeed, particles being on average smaller and made of cancellous bone only, like those of ABB, should remodel faster and not more slowly, as it is observed in the present study. Yet, as these are potentially confounding factors, such differences should be the subject of future studies.

How this different behavior affects the clinical outcome merits discussion. Ideally, bone grafts should remodel at a rate like that of the skeletal segment they are placed in, to provide enough mechanical support while new bone formation occurs [38,39]. This is true also concerning the mechanical properties of alveolar bone and the support it must provide to dental implants: residual graft particles, in fact, may influence the latter [40]. They also may interfere with the first biological events that lead to osseointegration [41]. Accordingly, EDEB might provide a higher probability of successful osseointegration. Yet ABB, showing a lower resorption rate than EDEB, could be more effective in preserving the bone volume of the grafted post-extractive sockets. This study does not provide any short or long-term assessment of alveolar bone resorption. Further comparative, prospective long-term studies should be carried out to specifically address this matter. Again, long-term prospective comparative studies should be performed to assess if the presence of a higher amount of newly-formed bone at implant placement, like that observed in the present study when EDEB was grafted, may result in higher long-term implant survival/success rates. Results of the present study show, within the limits of detection of the protein assay techniques used, that enzyme-deantigenic equine bone (EDEB) contains type I bone collagen in its native state, with no traces of collagen degradation products, while anorganic bovine bone (ABB) contains no collagen or other proteins. Enzyme-deantigenic equine bone (EDEB) allowed a significantly greater quantity of bone to form at the grafted sites than anorganic bovine bone (ABB).

In conclusion, the presence of native collagen within a bone xenograft may lead to greater and faster new bone formation than that achievable when a collagen-free, slow-resorbing xenograft is used. This may give the oral surgeon different options when carrying out bone regeneration for delayed implant placement, where possibly a collagen-preserving xenograft might be preferred, or for reconstructing aesthetic profiles, where possibly a collagen-free slow-resorbing xenograft might be the best option. In a condition like that found in regenerating a post-extractive socket for delayed implant placement, where both needs are present, i.e., that of placing the implant in vital newly-formed bone, and that of preserving alveolar bone levels which of the two options is best is still open to debate: how this choice can affect both long-term alveolar bone preservation and implant success and survival rates should be the subject of further prospective, controlled investigations.

## Figures and Tables

**Table 1:** Summary of data and results of the present study.

EDEB							ABB						
PT	Gender	Age	Socket	Time	NFB	RB	PT	Gender	Age	Socket	Time	NFB	RB
1	M	45	15	4.6	46.92	15.15	1	M	56	25	3.0	42.45	12.18
			16		42.16	16.06	2	M	45	45	3.9	35.60	13.26
2	F	56	35	3.2	44.46	8.98				46		38.01	24.05
			36		58.43	4.54	3	F	46	45	5.9	42.32	23.43
3	F	45	27	4.3	44.28	5.74				46		23.20	10.94
4	M	56	36	6.3	41.66	7.19				47		39.11	15.81
5	M	57	35	4.8	58.00	6.20	4	F	57	26	5.0	48.22	21.14
			36		30.11	19.44	5	M	58	35	5.8	46.44	19.14
6	F	43	35	2.7	40.61	15.15				36		27.18	20.74
7	F	57	25	4.5	27.78	13.40				37		21.69	13.29
8	F	56	46	4.0	57.30	10.77	6	M	46	15	3.4	20.67	24.40
			47		51.60	11.54	7	M	65	35	3.8	19.74	23.73
9	M	48	45	5.4	38.45	8.32				46		44.67	16.76
			46		34.74	5.65	8	F	45	25	3.6	32.39	27.04
10	M	65	35	3.5	31.41	8.23	9	F	48	35	3.8	37.77	10.91
11	F	56	36	2.9	37.75	8.14				36		44.36	13.70
			37		31.75	16.83	10	M	65	35	3.8	30.48	26.28
12	M	48	36	6.3	61.06	7.19				36		42.96	24.50
			37		60.97	5.67	11	F	73	45	6.0	45.33	27.70
13	M	55	36	5.1	40.82	12.27	12	M	54	45	4.5	33.82	16.54
			37		58.93	10.97				46		32.85	15.76
14	F	56	35	4.8	43.51	6.44	13	M	65	35	3.5	20.29	26.47
			36		38.77	6.18	14	M	55	45	4.2	31.60	22.69
15	F	45	21	3.1	42.36	10.47				46		41.15	20.67
16	M	46	45	4.9	59.16	7.36	15	F	56	36	4.3	45.79	18.31
			46		58.32	18.56				37		38.12	14.37
			47		39.17	19.66	16	M	67	11	4.7	38.07	17.62
17	M	56	35	2.9	33.36	8.10	17	F	48	34	2.7	24.22	18.65
			36		49.25	11.35				35		19.84	12.33
			37		62.04	16.66	18	M	56	46	2.9	19.58	24.94
18	M	46	35	5.4	57.17	15.65				47		22.85	14.40
			36		48.44	14.79				48		22.07	26.81
			37		56.42	14.59	19	F	46	21	5.1	20.40	20.10
19	F	56	22	5.2	37.57	11.12	20	M	44	13	4.7	29.90	13.46
20	F	57	45	2.8	50.58	8.25	21	F	58	45	5.2	41.89	11.28
			46		32.50	13.02				46		35.68	25.52
21	F	56	36	5.7	52.06	8.40	22	M	57	35	3.6	49.60	9.62
			37		29.31	5.90				36		21.11	8.87
22	M	58	36	5.4	38.51	9.10	23	M	56	21	3.4	46.05	10.65
			37		37.08	13.52	24	F	55	35	3.7	26.69	20.30
										36		33.91	18.84
**Mean**		52.9		4.1	45.12	10.91			55.0		4.4	33.61	18.47
**SD**		5.9		1.2	10.54	4.27			8.1		1.2	9.71	5.62
